# PIM2 Induced COX-2 and MMP-9 Expression in Macrophages Requires PI3K and Notch1 Signaling

**DOI:** 10.1371/journal.pone.0004911

**Published:** 2009-03-17

**Authors:** Kushagra Bansal, Nisha Kapoor, Yeddula Narayana, Germain Puzo, Martine Gilleron, Kithiganahalli Narayanaswamy Balaji

**Affiliations:** 1 Department of Microbiology and Cell Biology, Indian Institute of Science, Bangalore, India; 2 Department of Molecular Mechanisms of Mycobacterial Infections, Institut de Pharmacologie et de Biologie Structurale du Centre National de la Recherche Scientifique (CNRS) and Université Paul Sabatier, Toulouse, France; Institut Pasteur Korea, Republic of Korea

## Abstract

Activation of inflammatory immune responses during granuloma formation by the host upon infection of mycobacteria is one of the crucial steps that is often associated with tissue remodeling and breakdown of the extracellular matrix. In these complex processes, cyclooxygenase-2 (COX-2) plays a major role in chronic inflammation and matrix metalloproteinase-9 (MMP-9) significantly in tissue remodeling. In this study, we investigated the molecular mechanisms underlying Phosphatidyl-*myo*-inositol dimannosides (PIM2), an integral component of the mycobacterial envelope, triggered COX-2 and MMP-9 expression in macrophages. PIM2 triggers the activation of Phosphoinositide-3 Kinase (PI3K) and Notch1 signaling leading to COX-2 and MMP-9 expression in a Toll-like receptor 2 (TLR2)-MyD88 dependent manner. Notch1 signaling perturbations data demonstrate the involvement of the cross-talk with members of PI3K and Mitogen activated protein kinase pathway. Enforced expression of the cleaved Notch1 in macrophages induces the expression of COX-2 and MMP-9. PIM2 triggered significant p65 nuclear factor -κB (NF-κB) nuclear translocation that was dependent on activation of PI3K or Notch1 signaling. Furthermore, COX-2 and MMP-9 expression requires Notch1 mediated recruitment of Suppressor of Hairless (CSL) and NF-κB to respective promoters. Inhibition of PIM2 induced COX-2 resulted in marked reduction in MMP-9 expression clearly implicating the role of COX-2 dependent signaling events in driving the MMP-9 expression. Taken together, these data implicate PI3K and Notch1 signaling as obligatory early proximal signaling events during PIM2 induced COX-2 and MMP-9 expression in macrophages.

## Introduction

*Mycobacterium tuberculosis*, the causative agent of pulmonary tuberculosis, infects one-third of the world's population [1]. The granuloma formation represents pathological attributes of the host immunity to *M. tuberculosis* infection and is responsible for the containment of the infection. It has been suggested that the granuloma formation represents a complex process involving initiation and development of organized multicellular structures comprised of macrophages, CD4 and CD8 T cells, dendritic cells as well as components of extracellular matrix (ECM) [2]. Despite the well-documented pathological attributes of infection triggered granuloma formation, molecular details on the granuloma formation in terms of the role of inflammatory responses with reference to ECM proteins or lymphocytes trafficking are inadequately understood. Matrix metalloproteinases (MMPs) are Zn^2+^ and Ca^2+^ dependent endopeptidases which participate in a significant manner in several aspects of host immune responses such as granuloma formation, matrix remodeling, lymphocytes trafficking and infiltrations, inflammation etc. Among MMPs, MMP-9 is expressed at various clinical categories of tuberculosis disease like active cavitary tuberculosis [3]–[4], meningitis [5]–[6] and pleuritis [7]. In case of pulmonary tuberculosis, breakdown of ECM forms an integral part of the granuloma formation [8]. Mycobacterial species are known to induce MMP-9 expression and MMP-9 induction in macrophages is suggested to involve Cyclooxygenase-2 (COX-2) dependent signaling events [9]. COX-2 is a key enzyme that catalyzes the rate-limiting step in the inducible secretion of Prostaglandin E2 (PGE2) [10]. In this perspective, studies have suggested that MMP-9 expression in macrophages was induced by Prostaglandin E2, and inhibition of COX-2 resulted in inhibition of mycobacterium triggered MMP-9 expression [9]. Inhibition of macrophage COX-2 activity resulted in marked reduction in ECM induced expression of MMP-9 [11]. Further, in COX-2 null macrophages, MMP-9 expression was markedly reduced in comparison to wild type suggesting the role of COX-2-MMP-9 axis as significant factor at sites of chronic inflammation [11]–[12]. Taken together, COX-2 dependent PGE2 production appears to be an important factor in driving the MMP-9 expression, a step critical for the breakdown of the ECM components during formation of granulomas. In addition to many species of mycobacteria, the mycobacterial antigens are known to trigger the inducible expression of COX-2 and MMP-9 [9], [13]–[15].

Macrophages are principal mediators of initiation as well as activation of host inflammatory responses to tuberculosis infection. Albeit mycobacteria reside within phagolysosomes of the infected macrophages, envelope glycoconjugates like Lipoarabinomannan (LAM), phosphatidyl-*myo*-inositol mannosides (PIM), Trehalose 6,6′-dimycolate (TDM; cord factor) etc., are released and traffic out of the mycobacterial phagosome into endocytic compartments as well as can gain access to the extracellular environment in the form of exocytosed vesicles [16]–[17]. In this perspective, PIM represents a variety of phosphatidyl-*myo*-inositol mannosides (PIM) 1–6 containing molecules and are integral component of the mycobacterial envelope. A number of biological functions have been credited to PIM2 [18], [19]–[24]. PIM2 was shown to trigger TLR2 mediated activation of macrophages that resulted in activation of nuclear factor– κB (NF-κB), AP-1, and mitogen-activated protein kinases (MAPK) [25]. In addition to pulmonary granuloma-forming activities, PIM2 was shown to recruit NKT cells into granulomas [26]. Further, PIM was suggested to act as adhesins mediating attachment of *M. tuberculosis* bacilli to non-phagocytic cells [27]. Accordingly, mycobacterial envelope antigen PIM2 could initiate or affect the inflammatory responses similar to mycobacteria bacilli.

In the present study, we set out to delineate the signaling cascades regulating PIM2 triggered expression of MMP-9 and COX-2 in macrophages. Albeit MAPK and NF-κB signaling pathways are generally believed to be involved [28]–[30], little is known about the signaling molecules playing significant roles upstream of MAPK and NF-κB during mycobacterial antigens induced COX-2 and MMP-9 expression. Our current study provides the evidence that PIM2 driven activation of Notch and Phosphoinositide 3-kinase (PI3K) signaling cascades triggers the expression of COX-2 and MMP-9.

Among diverse signaling cascades, Notch signaling pathway is suggested to execute important function during initiation or activation of inflammatory immune responses [31]. In general, productive interaction of Notch receptor with its ligand causes the proteolytic cleavage mediated by gamma-secretase complex to release Notch Intra Cellular Domain (cleaved Notch or NICD). NICD then translocates to the nucleus and collaborate with DNA binding protein CSL/RBP-Jk along with coactivators leading to the transcription of its target genes [32]. On the other hand, it has been demonstrated that NICD can regulate the expression of many of its target genes in a transcription-independent manner by activating PI3K and MAPK signaling cascades [33]–[34]. The PI3K-AKT signaling cascade regulates and modulates several cellular processes including cell survival, proliferation, growth etc. [35]. Additionally, survival effects of Notch signaling are reported to be mediated by activation of the MAPK in many tumors and the regulation of PI3K-AKT-MAPK axis could offer a mechanistic basis for Notch signaling in the promotion of primary tumor progression [32].

TLR stimulation by various agonists was shown to activate Notch signaling resulting in modulation of diverse target genes involved in pro-inflammatory responses in macrophages [13], [36]–[37]. Further, we demonstrated previously that *M. bovis* BCG induced expression of SOCS3 involved Notch1 upregulation and activation of Notch1 signaling pathway in a TLR2-MyD88 manner [36]. However, possible implications of Notch signaling on immunological parameters associated with interaction of host macrophages with intracellular pathogen like mycobacteria or its novel cell wall antigens remain scanty.

In this study, we show that PIM2 triggered COX-2 and MMP-9 expression requires the involvement of PI3K and Notch1 signaling and distinctly, perturbation of Notch1 signaling suggests involvement of the cross-talk with members of PI3K and MAP kinase pathway. These data establish a role for PI3K and Notch1 signaling in PIM2 induced COX-2 and MMP-9 expression in macrophages.

## Results

### PIM2 induces COX-2 and MMP-9 expression in macrophages

**Figure 1:**
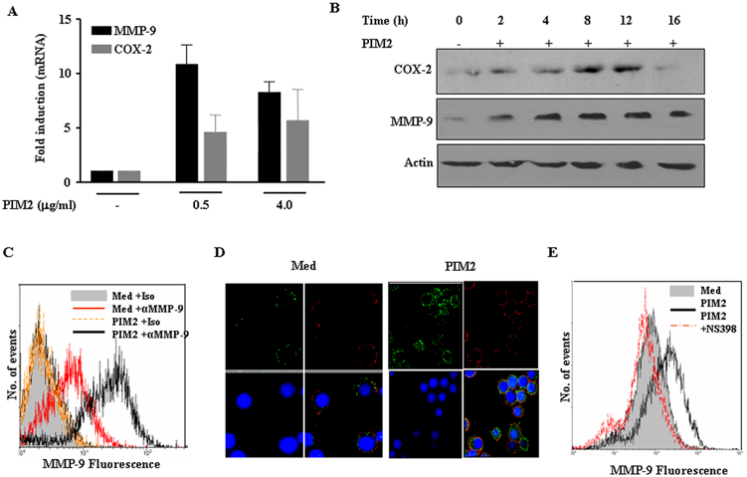
PIM2 induces expression of COX-2 and MMP-9 in mouse peritoneal macrophages. (A). Mouse peritoneal macrophages were treated with 0.5 and 4.0 µg/ml of PIM2 and mRNA levels of COX-2 and MMP-9 were analyzed by Quantitative real time PCR. (B). The levels of COX-2 and MMP-9 protein expression were evaluated by immunoblotting in total cell lysates prepared from macrophages treated with 4.0 µg/ml of PIM2 for different time points. (C). Flow cytometric analysis of MMP-9 expression on the surface of PIM2 treated macrophages. Cells were probed with anti-MMP-9 or isotype matched control antibody followed by anti- rabbit FITC. (D). Immunoflourescent staining of MMP-9 on macrophages treated with 4.0 µg/ml of PIM2 as analyzed by confocal microscopy. Cells were fixed and MMP-9 expression was detected by binding of specific or isotype matched antibodies followed by probing with Cy-2 (green) labeled anti-rabbit secondary antibody. Nucleus of macrophages were stained with nuclear staining dye, Hoechst 33342 (blue) and plasma membrane with anti-MHC Class I antibody-Cy5 (red). (E). Mouse macrophages were cultured with or with out NS-398 (10 µM) and treated with 4.0 µg/ml of PIM2 for 12 h. The protein levels of MMP-9 were analyzed by immunoflourescent staining of MMP-9 followed by flow cytometry. The data represented in the figure are representative of three independent experiments. *Med*, Medium.

In this study, we examined the molecular details of PIM2 induced signaling events that are leading to the expression of COX-2 and MMP-9. PIM2 treatment of mouse peritoneal macrophages triggers the expression of COX-2 and MMP-9 both at mRNA and protein levels (Figure 1A–B). PIM2 triggered expression of both COX-2 and MMP-9 transcripts as early as 2 hours, clearly peaking in their expression at 12 hrs (Figure S1A). Accordingly, PIM2 induced COX-2 and MMP-9 protein expression could be detected as early as 2 hours (faint band) up to 16 hours of the post-PIM2 treatment (Figure 1B). Further, in addition to the release of MMP-9 in the medium, experiments were carried out to explore whether PIM2 increases the cell membrane associated MMP-9 by flow cytometry. PIM2 treatment increased the percentage of cells that were stained positive for membrane MMP-9 compared to untreated cells (Figure 1C). Confocal microscopy studies further validated PIM2 triggered increased cell-surface association of MMP-9, where in macrophages were stained for nucleus (with nuclear staining dye, Hoechst 33342, blue), plasma membrane (with anti-MHC Class I antibody, red) and for MMP-9 (with anti-MMP-9 antibody, green). The merged images from confocal microscopic analysis revealed that MMP-9 or MHC class I do not colocalize clearly demonstrating a distinct cell-surface association of MMP-9 (Figure 1D and Figure S1B). COX-2 is known to regulate mycobacteria triggered MMP-9 expression and accordingly, when tested, COX-2 inhibitor NS-398 markedly blocked MMP-9 expression both at RNA (Figure S2A) and protein levels clearly implicating the role COX-2 dependent signaling events in PIM2 induced MMP-9 expression (Figure S2B). In order to further ascertain the specific effect of NS-398, we analyzed the cell-surface expression of MMP-9 upon PIM2 treatment in the presence or absence of NS-398. Flow cytometry results presented in Figure 1E demonstrate that blocking COX-2 activity by NS-398 significantly reduced PIM2 triggered surface MMP-9 expression. Further, purified PGE2 increased MMP-9 expression in a dose dependent manner in mouse macrophages (Figure S3). In order to exclude the autocrine or secondary effect of macrophage produced cytokines on PIM2 triggered COX-2 and MMP-9 expression, mouse macrophages were treated with PIM2 in the presence or absence of cycloheximide. The data presented clearly demonstrates that PIM2 triggered COX-2 and MMP-9 mRNA expression was not affected by cycloheximide clearly ruling out the autocrine effect of PIM2 induced cytokines on COX-2 and MMP-9 expression (Figure S4A–B). However, interestingly, cycloheximide augmented PIM2 triggered COX-2 mRNA expression. Extensive literature survey suggests that the regulation of COX-2 gene is complex and transcription of COX-2 is reported to vary across the cell types as well as among responses of a given cell type to variety of stimuli [54]–[56]. This effect has been attributed to the presence of AU-rich elements in 3′-untranslated region of COX-2 mRNA, which is suggested to be an important determinant in COX-2 mRNA stability in human macrophages [55] as well as in diverse cell types [54].

### PIM2 triggers activation of PI3 kinase signaling pathway

**Figure 2:**
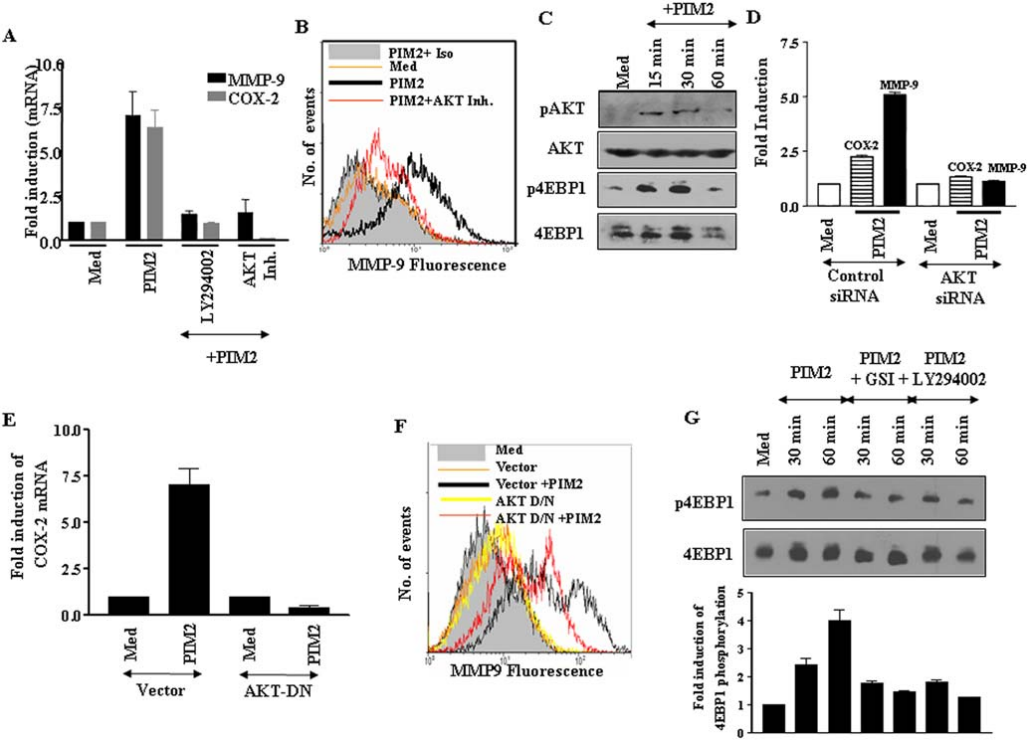
PIM2 induced upregulation of COX-2 and MMP-9 requires Notch1 mediated activation of PI3 Kinase pathway. (A). COX-2 and MMP-9 transcript levels were analyzed in mouse macrophages pretreated with LY294002 (50 µM), AKT inhibitor II (10 µM) or 0.1% DMSO as vehicle control and then cultured in the presence of PIM2 (4.0 µg/ml) for 12 h. (B). MMP-9 at the cell surface was analyzed by flow cytometry on PIM2 treated macrophages cultured with or without AKT inhibitor II (10 µM) or 0.1% DMSO as vehicle control. (C). Kinetics of phosphorylation of AKT and 4EBP1 upon treatment of PIM2. The blots were stripped and probed for total levels of AKT and 4EBP1 as control. (D). RAW 264.7 macrophages were transiently transfected with either control siRNA or AKT siRNA. After three days of transfection, cells were treated with PIM2 and protein levels of AKT as well as COX-2 and MMP-9 were analyzed. The blots from three independent experiments were densitometrically quantitated and represented as fold change over medium. (E). RAW 264.7 macrophages were transfected with either empty vector or dominant-negative AKT (AKT-DN) followed by treatment with PIM2. Transcript levels of COX-2 were analyzed by quantitative real-time PCR and (F). Surface expression of MMP-9 was analyzed by flow cytometry using rabbit anti-MMP-9 antibody followed by probing with Cy-2 labeled anti-rabbit secondary antibody. (G). Mouse macrophages were pretreated with LY294002 (50 µM) or GSI-I (10 µM) and activation of 4EBP1 at 30 and 60 min post treatment of PIM2 was analyzed. Histogram represents densitometric analysis of phospho-4EBP1 normalized against total 4EBP1 derived from three independent experiments. The results shown represent three independent experiments. *Med*, Medium.

Mycobacteria are known to trigger the activation of intracellular signalling pathways [38], such as PI3K and MAPK including extracellular signal-regulated kinase (ERK) 1/2, p38 kinase as well as stress-activated protein kinase/c-Jun NH2-terminal kinase (SAPK/JNK). In view of published observations [38], attempts were made in the current investigation to elucidate the role of PI3K and MAPK in PIM2 triggered expression of COX-2 and MMP-9. We used pharmacological inhibitors of PI3K, AKT and MAPK to test the functional involvement of the PI3K or MAPK signaling in PIM2 treated macrophages. In this context, Figure 2A clearly demonstrates that inhibitors of PI3K, LY294002 or AKT inhibitor, clearly reversed PIM2 triggered COX-2 and MMP-9 expression. Additionally, AKT inhibitor markedly decreased PIM2 induced surface expression of MMP-9 as well as COX-2 protein levels (Figure 2B and data not shown). In addition, PI3K inhibitor, LY294002, abrogated PIM2 triggered cell-surface expression of MMP-9 (Figure S5). Further, PIM2 triggered phosphorylation of AKT and 4EBP1 (Figure 2C) clearly demonstrating the activation of PI3K pathway. Perturbation of PI3K signaling cascade by siRNA to AKT (Figure S8A) resulted in striking reduction in COX-2 and MMP-9 expression (40% and 81% decrease for COX-2 and MMP-9 to actin protein ratio respectively, Figure 2D). Further, AKT dominant negative construct effectively blocked PIM2 ability to induce COX-2 (Figure 2E) and MMP-9 expression (Figure 2F).

**Figure 3:**
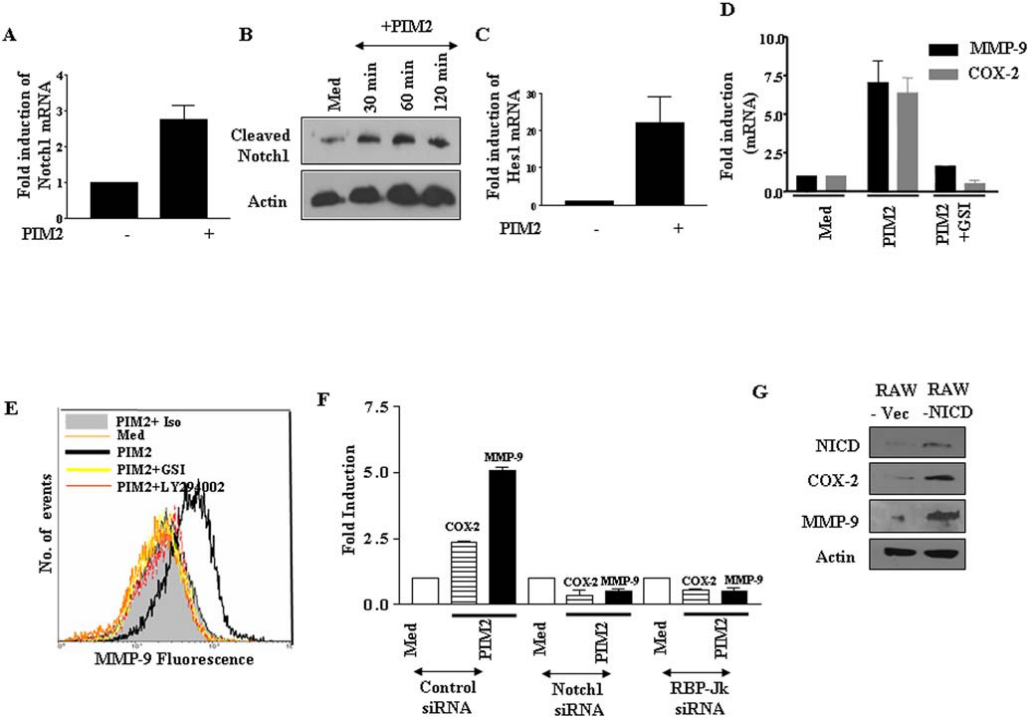
PIM2 induced expression and activation of Notch1 is involved in induced expression of COX-2 and MMP-9 in macrophages. (A). Real-time PCR quantification of Notch1 mRNA expression levels in mouse macrophages after treatment with 4.0 µg/ml of PIM2 for 12 h (B). Kinetics of protein expression of Cleaved Notch1 was analyzed in mouse macrophages treated with 4.0 µg/ml of PIM2 for the indicated time points. (C). Hes1 transcript levels were assessed by real time PCR in mouse macrophages after treatment with 4.0 µg/ml of PIM2 for 12 h. (D). RAW 264.7 macrophages stably transfected with pCMV-NICD, Notch intracellular domain, (RAW-NICD) or vector pCMV (RAW-Vec) were analyzed for the expression of NICD, COX-2, MMP-9 and β-actin as loading control. (E). PIM2 (4.0 µg/ml) treated macrophages were cultured with or with out γ-secretase inhibitor GSI-I (10 µM) and the levels of COX-2 and MMP-9 transcript (at 12 h) were analyzed by quantitative real time PCR. (F). Macrophages were treated with 4.0 µg/ml of PIM2 for 12 h cultured in the presence of LY294002 (50 µM), GSI-I (10 µM) or 0.1% DMSO and cell surface expression of MMP-9 was analyzed by flow cytometry. (G). RAW 264.7 macrophages were transfected with 100 nM of small interfering RNA (siRNA) directed to either Notch1 or RBP-Jk siRNA or control siRNA. Three days post transfection, cells were treated with PIM2 for 12 h and protein levels of COX-2 as well as MMP-9 were analyzed. The blots from three independent experiments were quantitated by densitometry and represented as fold change over medium. The results are expressed as mean±SEM of three independent experiments and the blots are representative of three independent experiments. *Med*, Medium.

In order to further delineate the signaling events, peritoneal macrophages were treated with LY294002, a PI3K inhibitor before PIM2 treatment. The inhibitor LY294002 significantly reduced PIM2 activated 4EBP1 phosphorylation (Figure 2G). During this stage of current investigation, experimental results obtained in parallel suggested the involvement of Notch1 signaling in PIM2 induced COX-2 and MMP-9 expression. These results are presented in detail in Figure 3 of this manuscript. Since it has been suggested that PI3K-AKT pathway can operate in concert as a gain control for Notch signal responses in many cell types, attempts were carried out to assess whether Notch activation inhibitor, γ-secretase inhibitor-I (GSI-I) could abrogate PIM2 triggered phopshorylation of AKT, mTOR, and 4EBP1. GSI-I, when tested, significantly blocked PIM2 triggered AKT, mTOR and 4EBP1 (Figure S6 and Figure 2G). These findings implicate possible cross-talks between PI3K and Notch signaling cascades during PIM2 induced expression of COX-2 and MMP-9.

### PIM2 mediated activation of PI3K signaling involves Notch activation

As described, many studies have suggested that PI3 kinase and MAPK pathways induced survival effects in many cell types often involve Notch1 signaling [32]–[34]. In this regard, experiments were carried out to assess whether PIM2 can trigger Notch expression and as represented in Figure 3A, PIM2 induced the upregulation of Notch1, both at mRNA and at protein levels as quantified by real-time PCR and western blot analysis respectively (data not shown). Further, PIM2 treatment enhanced the expression of Notch ligands, Jagged and Delta as well as expression of different members of Notch family like Fringes, Presinilin and Kuzbanian (Figure S7 and data not shown). Additionally, immunoblotting experiments demonstrated a similar level of induction of Notch1 intracellular domain (NICD), a cleavage product of Notch1 as well as full-length Notch1 in macrophages upon treatment with PIM2 (Figure 3B and data not shown). Further in agreement with Notch activation, the Notch1 target gene Hes1 [32] was also induced by PIM2 treatment (Figure 3C). Furthermore, we generated RAW 264.7 stable transfectants constitutively expressing Notch1 intracellular domain (RAW-NICD). The constitutively active NICD protein induced COX-2 and MMP-9 expression as well as the expression of Notch target gene, Hes1 (Figure 3D and data not shown). In addition, PIM2 further augmented ability of constitutively active NICD to induce COX-2 and MMP-9 expression (data not shown). These results evidently strengthen the role of possible crosstalk between PI3K and Notch signaling during PIM2 induced expression of COX-2 and MMP-9.

In order to further validate the involvement of upregulated Notch1 or NICD, chemical inhibitors that affect the Notch processing were tested for their effect on COX-2 and MMP-9 expression. As shown in Figure 3E–F, GSI-I, γ-secretase inhibitor blocked PIM2 triggered COX-2 and MMP-9 expression. In addition to GSI-I, the PI3K inhibitor, LY294002 markedly inhibited PIM2 triggered cell-surface associated MMP-9 as well as secreted form of MMP-9 (Figure 3F, Figure S5 and data not shown) implicating the role of both Notch and PI3K signaling in PIM2 triggered signaling events. In these experiments, treatment with the GSI-I resulted in a loss of processed Notch1-NICD (data not shown), which preceded the induction of COX-2 and MMP-9 expression. This was accompanied by a minimal change if any in expression of total Notch1 (data not shown). Further, siRNA mediated inhibition of Notch1 signaling (Figure S8B) resulted in marked reduction in COX-2 and MMP-9 expression (Figure 3G). Evidence in the literature suggests that multiple effects of Notch signaling often require both transcription-dependent and transcription-independent processes [32], [39]. In this perspective, a potential role of CSL/RBP-Jk in Notch1 induced expression of COX-2 and MMP-9 was investigated. The activation of Notch target genes often involves interaction of NICD with DNA binding protein, CSL/RBP-Jk. In order to address the effect of Notch1 signaling on CSL/RBP-Jk activation, RAW 264.7 macrophages were transfected with Hes1-Luc, followed by treatment with PIM2 either in the presence or absence of GSI-I, a Notch1 activation inhibitor. The data presented in Figure S9 demonstrates that the inhibition of Notch1 signaling significantly blocks the CSL/RBP-Jk dependent activation of Notch target gene, Hes1 gene transcription. Further, the downregulation of CSL/RBP-Jk by siRNA (Figure S8C) in PIM2 treated macrophages resulted in significant decrease in COX-2 and MMP-9 expression (97% and 87% decrease in COX-2 and MMP-9 to actin protein ratio respectively, Figure 3G). This result clearly suggests that Notch signaling regulates COX-2 and MMP-9 expression to a significant extent in a transcription-dependent manner.

**Figure 4:**
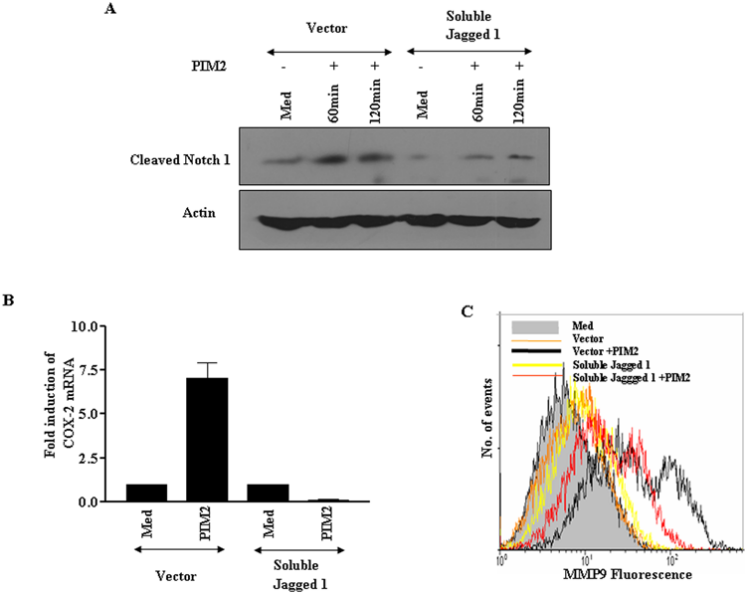
Soluble Jagged 1 significantly inhibits PIM2 triggered cleaved Notch1 (NICD), COX-2 or MMP-9 expression. (A). Cleaved Notch1 levels were assessed in RAW 264.7 macrophages transfected either with soluble Jagged1 construct or Vector construct followed by treatment with PIM2. (B). As described in (A), in soluble Jagged1 construct transfected cells, transcript levels of COX-2 were analyzed by quantitative real-time PCR and (C). surface expression of MMP-9 by flow cytometry. The results presented are representative of three independent experiments. *Med*, Medium.

Further, in addition to siRNA and pharmacological inhibition of Notch1 signaling, we have made use of soluble form of Jagged1, a known Notch1 ligand, to inhibit PIM2 triggered Notch1 signaling. Many studies have reported that, when expressed, extracellular soluble form of Jagged1 exerts dominant-negative effect on Notch signaling possibly by sequestering the Notch receptor and preventing Notch interaction with its ligands [40]–[42]. In this perspective, soluble Jagged1 markedly reduced not only PIM2 triggered cleaved Notch1 (NICD) formation (Figure 4A), but also the subsequent expression of COX-2 and MMP-9 in macrophages (Figure 4B–C).

### Involvement of TLR2 in PIM2 triggered Notch1 signaling

**Figure 5:**
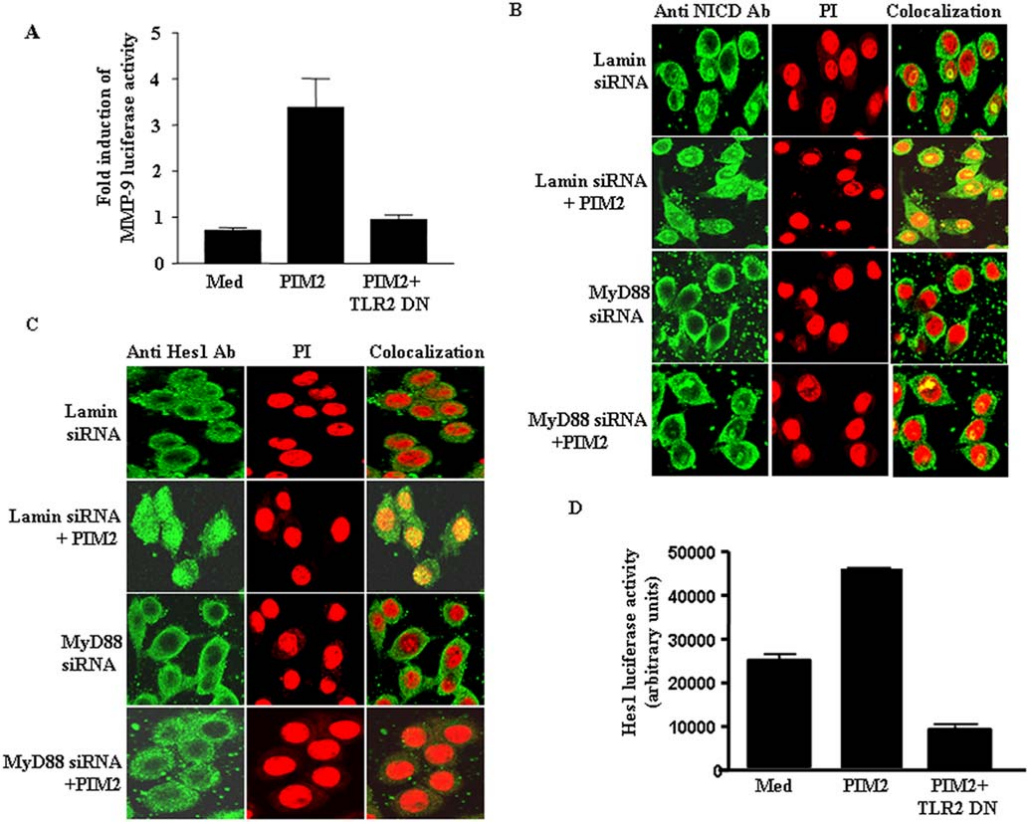
TLR2-MyD88 axis plays important role in PIM2 triggered Notch1 activation. (A). TLR2 dominant-negative construct significantly reduced PIM2 triggered MMP-9 expression as assayed by MMP-9 luciferase reporter activity as described in Materials and Methods. (B). siRNA mediated downregulation of MyD88 in PIM2 triggered nuclear localization of NICD as well as (C). in expression of Notch1 target gene, Hes1, as analyzed by confocal microscopy. (D). RAW 264.7 macrophages were transfected with either vector or dominant-negative TLR2 (TLR2-DN) and Hes1-Luc, followed by treatment with PIM2. The Hes1 promoter activity was evaluated by Luciferase assay. Data presented in the figure is representative of three independent experiments. *Med*, Medium.

Studies have suggested that mycobacteria induced the expression of COX-2 or MMP-9 required the involvement of TLR2 [9] and PIM2 triggered secretion of proinflammatory stimuli such as TNF-α and IL-12 in a TLR2 dependent manner in murine and human macrophages [24]. In this context, when assessed, TLR2 dominant-negative construct significantly reduced PIM2 triggered MMP-9 expression (Figure 5A) clearly implicating the role of TLR2 in PIM2 mediated activation of signaling events in macrophages. Further, siRNA mediated downregulation of MyD88 expression; a downstream adaptor molecule among majority of TLR signaling pathways, resulted in substantial reduction in PIM2 induced MMP-9 expression (Figure S10).

In order to further ascertain the role of TLR2 pathway in Notch1 activation, RAW264.7 macrophages were either transfected with siRNA targeted to MyD88 or with control siRNA followed by PIM2 treatment. The data presented in Figure 5B clearly demonstrates that knockdown of MyD88, a downstream adaptor molecule in TLR signaling pathways, resulted in the inhibition of the expression and nuclear localization of NICD. Further, MyD88 siRNA, but not control siRNA blocked the nuclear expression of Hes1 (Figure 5C), a marker gene for Notch1 activation. In order to assess localization of NICD or Hes1 in nucleus, a series of images at an interval of 0.37-mm focal planes were collected into a z-stack. Every single layer of z-stack was subjected to image analysis by LSM 5 image examiner software to visualize and locate NICD/Hes1 protein expression in the nucleus. Figure S11A demonstrates PIM2 triggered translocation of NICD (green) or Hes1 (green) into nucleus (red). The truly colocalized areas/spots exhibited a yellow color (the non-overlapping spots were either red or green). In addition, scatter plot were also created using software Combi-FCS LCM META, which were utilized for obtaining correlation coefficient, a measure of the strength relationship between the two signals as well as a measure of how well two signals are correlated (Figure S11B–C).

Additionally, inhibition of TLR2 signaling by TLR2 dominant-negative construct resulted in substantial reduction in Hes1-promoter activity (Figure 5D). These data are in complete agreement with the published studies where in TLR2-mediated Notch1 up-regulation relied completely on the MyD88 adaptor protein and TLR2 but not TLR4-mediated, Notch1 up-regulation was completely abrogated in MyD88^−/−^ macrophages [37].

### PIM2 triggered COX-2 and MMP-9 expression involves activation of ERK1/2

**Figure 6:**
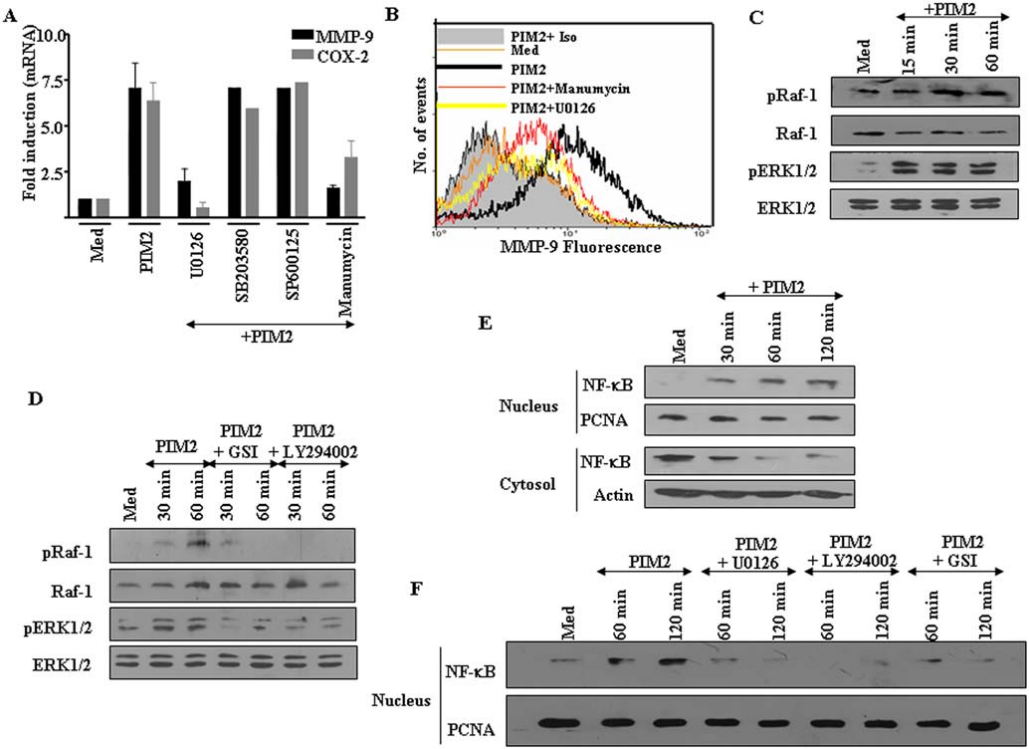
PIM2 induced activation of ERK1/2 and NF-κB is dependent on Notch1 mediated activation of PI3 kinase pathway. (A). Mouse macrophages were treated with U0126 (10 µM), SB203580 (20 µM), SP600125 (50 µM), Manumycin (20 µM) or DMSO as vehicle control, prior to treatment with PIM2 for 12 h and COX-2 and MMP-9 mRNA expression levels were analyzed by real time PCR. (B). MMP-9 levels on the cell surface were assessed by flow cytometry on PIM2 treated macrophages pretreated with Manumycin (20 µM), U0126 (10 µM) or DMSO. (C). Time dependent activation of Raf-1 and ERK1/2 upon treatment macrophages with 4.0 µg/ml of PIM2. (D). Mouse peritoneal macrophages were pretreated with LY294002 (50 µM) or GSI-I (10 µM) and phosphorylation status of Raf-1 and ERK1/2 at indicated time points after treatment with PIM2 was assessed. (E). Mouse macrophages were treated with PIM2 for indicated time points and were fractionated into cytosolic, nuclear fractions and probed for NF-κB by immunoblotting. (F). Nuclear translocation of NF-κB upon treatment of macrophages with PIM2. Macrophages were treated with U0126 (10 µM), LY294002 (50 µM), GSI-I (10 µM) prior to PIM2 treatment. The graphs show the average of three independent experiments±SEM. Representative blots from three independent experiments are shown. *Med*, Medium.

Consistent with signaling events reported in many cell types, Mycobacteria triggered PI3K signaling frequently reported to involve activation of other intracellular signaling cascades such as MAPK [38]. In this context, we were interested in assessing whether PIM2 triggers activation of MAPK, in addition to PI3K and Notch signaling. Hence, when tested, only U0126 (ERK1/2 inhibitor), but not SB203580 (p38 MAPK inhibitor) or SP600125 (JNK inhibitor) reduced PIM2 triggered COX-2 and MMP-9 expression (Figure 6A). In addition, Ras activation inhibitor, Manumycin clearly abrogated PIM2 induced COX-2 and MMP-9 expression (Figure 6A–B). Furthermore, exposure of mouse peritoneal macrophages to PIM2 resulted in an increase in the phosphorylation of Raf-1, one of the target proteins for Ras, and ERK1/2 apparent within minutes of PIM2 treatment of macrophages (Figure 6C). However, the COX-2 or MMP-9 expression level remained unperturbed when PIM2 triggered activation of p38 was inhibited (Figure 6A). Further, in order to delineate whether activation of ERK1/2 or Raf-1 by PIM2 involves PI3 Kinase or Notch1 signaling, the phospshorylation of ERK1/2 or Raf-1 by PIM2 was assessed in the presence of LY294002, an inhibitor of PI3K or GSI-I, γ-secretase inhibitor. PIM2 triggered activation of ERK1/2 or Raf-1 was markedly reduced, when PI3K signaling or activation of Notch was inhibited (Figure 6D). These results suggest that ERK1/2 represents as important mediator in the Notch1-PI3K signaling pathway by which PIM2 induces COX-2 and MMP-9 expression. Further, MyD88 is a known critical downstream adaptor molecule in TLR signaling and siRNA to MyD88 reduces Notch 1 signaling and PIM2 triggered MMP-9 expression (Figure 5B–C and Figure S10). In this context, we analyzed the effect of siRNA to MyD88 on PIM2 triggered ERK1/2 activation. As presented in Figure S12, siRNA to MyD88 abrogated PIM2 induced activation of ERK1/2 suggesting TLR2-MyD88 dependent PI3K-AKT-MAPK axis could be the basis for Notch signaling.

In order to further ascertain PIM2 ability to trigger Notch1 or COX-2 or MMP-9 expression, we attempted to inhibit PIM2 synthesis in *M. bovis* BCG by D-mannosamine, an inhibitor of PIM biosynthesis. Biochemical and genetic characterization of the *pimB* gene from *M. tuberculosis* have suggested that PimB is the α-D-mannose-α(1→6)-phosphatidyl-*myo*-inositol monomannoside transferase responsible for the formation of triacyl-PIM2 from GDP-Man and triacyl-PIM1. In this regard, investigators have extensively utilized D-mannosamine, an inhibitor of PIM biosynthesis, as a tool, to identify α-mannosyltransferases involved in PIM biosynthesis [43]–[44]. In this regard, when tested, blocking PIM2 synthesis in *M. bovis* BCG markedly reduced not only the formation of cleaved Notch1 (NICD), but also nuclear translocation of NICD in macrophages (Figure 13A & data not shown). Importantly, D-mannosamine treated *M. bovis* BCG fails to trigger the nuclear localization of Hes1, a Notch1 target gene product as well as the induced expression of COX-2 and MMP-9 (data not shown & Figure S13A–B). Even though, D-mannosamine did not affect the cell-viability or the overall colony morphology of the treated *M. bovis* BCG (data not shown), experiments were carried out to assess the cell wall composition of D-mannosamine treated *M. bovis* BCG in order to validate the above-mentioned results. Data presented in Figure S13C clearly demonstrates that treatment of *M. bovis* BCG with D-mannosamine resulted in marked reduction in PIM2 presence in *M. bovis* BCG, which in our opinion ascertains above-mentioned results.

### Involvement of NF-κB in PIM2 triggered COX-2 and MMP-9 expression

PI3K or Notch1 mediated activation of its target genes involves the active recruitment of transcription factor NF-κB [45]–[46]. Further, the promoters of COX-2 and MMP-9 are found to contain many *cis*-acting elements among which, NF-κB have been implicated in the regulation of both COX-2 and MMP-9 gene expression. In this context, PIM2 triggered significant p65 NF-κB translocation from the cytosol to the nucleus which peaked at 15–60 min and then decreased at 120 min of treatment (Figure 6E and data not shown). However these treatments did not alter the total PCNA (nucleus) or β-actin (cytosol) protein levels in the cells (Figure 6E). Additionally, with regard to cell fractionation studies, assessment of purity of the nuclear fraction was carried out using marker protein proliferating cell nuclear antigen (PCNA). In this context, the cytosol fractions were routinely checked for the presence of PCNA by immunoblotting to exclude the possibility of nuclear fraction contamination. PIM2 induced nuclear translocation of NF-κB was further confirmed utilizing the antibody specific for the phospho-p65 subunit of NF-κB (Figure S14).

The nuclear translocation of NF-κB, as shown in Figure 6E–F, is dependent on activation of Notch, as treatment with GSI-I, γ-secretase inhibitor, significantly reduced the nuclear translocation of NF-κB upon PIM2 treatment. Further, inhibitors of PI3K, LY294002 or ERK1/2, U0126 reversed PIM2 mediated nuclear translocation of NF-κB from cytosol (Figure 6F). In addition, an IkappaB phosphorylation inhibitor, BAY 11-7082 markedly diminished PIM2 induced MMP-9 and COX-2 protein levels (data not shown).

### PIM2 increases cell membrane association of MMP-9 via its interaction to CD44

**Figure 7:**
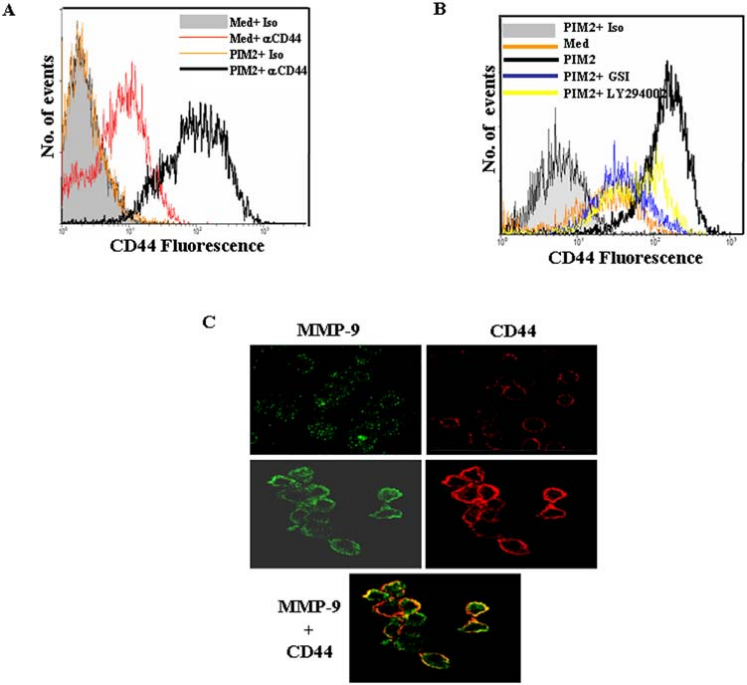
Association of PIM2 triggered cell surface MMP-9 associates with CD44. (A). Mouse macrophages were treated with PIM2 for 12 h and cell surface levels of CD44 were analyzed by probing with anti-CD44 or isotype matched control antibody followed by Cy5-conjugated goat anti- rat IgG. (B). CD44 levels on the cell surface were assessed by flow cytometry on PIM2 treated macrophages pretreated with LY294002 (50 µM) or GSI-I (10 µM). (C). Surface expression of MMP-9 and CD44 were evaluated by immunofluorescent staining using confocal microscopy. After 12 h treatment with PIM2, macrophages were probed with anti MMP-9 followed by anti rabbit IgG-Cy-2(green) as well as anti CD44 followed by anti- rat IgG-Cy5 (red). Merged image shows the areas of colocalization in yellow. The results are representative of three independent experiments. *Med*, Medium.

Many studies have suggested that MMP-9 associates with the cell membrane and the binding of MMP-9 is suggested to be mediated by several proteins including CD44 [47]. In this context, PIM2 increases the expression of cell surface associated MMP-9 as well as CD44 (Figure 1C and Figure 7A). PIM2 triggered cell surface CD44 expression can be blocked by inhibitors of PI3K (LY294002) or by Notch activation inhibitor, GSI-1 (Figure 7B). Further, for localization studies, double immunofluorescence experiments were carried out on PIM2 treated non-permeabilized macrophages (Figure 7C). The MMP-9 protein expression is indicated by green color and the red color indicates CD44 protein staining. The merged images from confocal laser scanning microscopic analysis revealed orange-yellow color shift indicating the co-localization of MMP-9 and CD44 proteins at the cell surface. The unstimulated cells exhibited weak signals of MMP-9 expression when stained either with isotypic or anti-MMP-9 antibody.

## Discussion

Activation of inflammatory immune responses by the host upon infection of pathogenic microbes is one of the crucial steps not only in controlling the spread of pathogen from the site of infection to reminder of host organs, but also in mounting an effective memory response so that future exposures/infections by similar pathogen/s can be effectively controlled. In this context, inflammatory responses during formation of granuloma in which mycobacteria resides is often associated with tissue remodeling and breakdown of the extracellular matrix. In these complex processes, both COX-2 and MMP-9 play a major role in chronic inflammation in general and MMP-9 significantly in tissue remodeling. Matrix metalloproteinases in general can degrade most of the components of ECM and among MMP's, MMP-9 is suggested to have important role in the pathogenesis of the tuberculosis disease [4]–[8]. During mycobacteria infection, MMP-9 induction has been implicated in cells recruitment, representing as an obligatory step in the formation of granuloma. However, MMP-9 expression can act as causative for the loss of the structural integrity of the lung tissues during chronic inflammation [8]. For an example, in case of tuberculous meningitis, increased MMP-9 expression is linked to tissue destruction, a pathological hallmark of inflammatory host immune response [4]–[5]. Further, evidences suggest that COX-2 dependent PGE2 regulates macrophage MMP-9 expression and COX-2 expression is induced in response to a variety of inflammatory mediators [12].

The current study attempts to delineate the signaling events that are involved in PIM2 mediated expression of COX-2 and MMP-9. We show that PIM2, an integral component of the mycobacterial envelope, induces the expression of MMP-9 as well as COX-2 in macrophages. We present the evidence that PIM2 triggered expression of COX-2 and MMP-9 involves the activation of PI3K and Notch1 signaling. Perturbation of Notch1 signaling by siRNA to Notch1 or pharmacological inhibition of Notch1 signaling or soluble form of Jagged1, which is suggested to exert dominant-negative effect on Notch1 activation [40]–[42], markedly inhibited PIM2 triggered Notch1 signaling as well as COX-2 and MMP-9 expression (Figure 3–4). Additionally, inhibition of COX-2 expression results in marked reduction in PIM2 triggered MMP-9 induction (Figure 1) clearly implicating the suggested role of COX-2 dependent signaling events in driving the MMP-9 expression [9]. In addition, enforced expression of the Notch1 intracellular domain (NICD) in RAW 264.7 macrophages induces the expression of both COX-2 and MMP-9 (Figure 3). Overall, our studies suggest that PI3K and Notch1 signaling contributes as an obligatory early proximal signaling event during PIM2 induced COX-2 and MMP-9 expression in macrophages. It is well known that TLRs significantly participate in wide variety of immune responses and are suggested to play pivotal role in the activation of inflammatory immune responses by mycobacteria. In this context, knockdown of MyD88, a downstream adaptor molecule in TLR signaling pathways resulted in inhibition of the expression and nuclear localization of Notch1 as well as nuclear expression of Hes1, a marker gene for Notch1 activation (Figure 5). The perturbation of TLR2 signaling by TLR2 dominant-negative construct also resulted in substantial reduction in Hes1 promoter activity (Figure 5). Additionally, our recent findings suggest that *M. bovis* BCG whole bacilli upregulates Notch1 and activates Notch1 signaling pathway in a TLR2-MyD88 dependent manner [36]. Our report clearly suggested that TLR2 can physically associate with both PI3K and Notch1, which implicates TLR2 triggering by *M. bovis* BCG in the activation of PI3K and Notch1. In this context, while PIM2 is suggested to stimulate murine and human macrophages in a TLR2 dependent manner [24], it is possible that PIM2 might trigger Notch1 signaling by interaction with TLR2. However, further investigations are required to expand the understanding of the above finding.

As mentioned in the introduction, the involvement of cross-talk between Notch and PI3K-AKT signaling cascades have been attributed to the overall cellular responses in diverse sets of cell types including CHO cells, primary T-cells and hippocampal neurons [48]. In addition, data suggesting the physical association of Notch1 with p56lck and PI3K in primary T cells as well as Notch mediated activation of the MAPK in many tumors suggest that regulation of PI3K-AKT -MAPK axis may be important component for Notch signaling [33]. In addition, transformation of many tumors by Notch requires active signals from the ERK/MAP kinase and PI3 kinase pathways along with Ras pathway [49]. Overall, these reports have suggested involvement of PI3K-AKT, ERK 1/2, and NF-κB, as possible downstream regulators of Notch signaling. In agreement with these reported findings, the inhibition of Notch1 or PI3K signaling resulted in significant decrease in phosphorylation of 4EBP1 (Figure 2), Raf-1 and ERK1/2 (Figure 6) clearly implicating PIM2 mediated activation as well as possible cross-interactions between Notch, PI3K and Ras/MAPK signaling molecules. Moreover, our data from inhibition of Notch1 or PI3K signaling intermediates by pharmacological inhibitors or dominant negative constructs suggest the possible positive regulation of PI3K-AKT and MAPK pathway by Notch1. Further, inhibition of PIM2 synthesis markedly compromises *M. bovis* BCG ability to trigger Notch1 or COX-2 or MMP-9 expression (Figure S13). These results clearly indicate that PIM2 is an important cell wall antigen that contributes to *M. bovis* BCG ability to trigger Notch signaling in macrophages.

Additionally, PIM2 treatment increases the surface expression of CD44 which associates with enhanced cell-surface MMP-9 (Figure 7). CD44 is shown to associate with a proteolytic form of MMP-9 on the surface of various tumor cells (47). Further, disruption of CD44/MMP-9 cluster formation had resulted in reduction of tumor invasiveness *in vivo* [50]–[51]. In this perspective, it had been postulated that CD44/MMP-9 complex formation on the cell surface might signify as a novel motility-enhancing signal for tumor cells which eventually contributes their invasiveness. Similar to results with MMP-9 expression, inhibitors of PI3K or Notch1 activation, LY294002 (PI3K) or GSI-I abrogated PIM2 mediated expression of CD44 (Figure 7).

**Figure 8:**
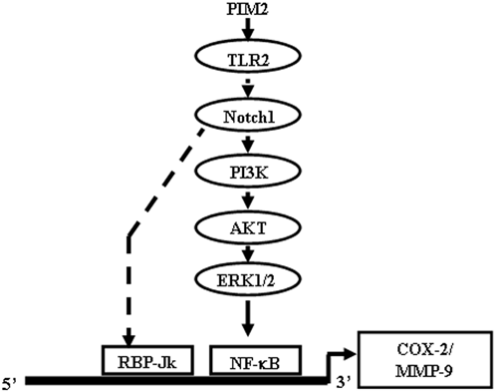
Model depicting PIM2 triggered signaling cascades regulating COX-2 and MMP-9 gene expression.

The PI3K or Notch1 signaling mediated activation of a given target gene often requires recruitment of transcription factor NF-κB [32]. Even though, the details of the PI3K mediated activation of NF-κB are reported, molecular understanding of precise interaction between Notch and NF-κB are not clear. However, recent reports suggest that Notch1 in addition through cross-talk with PI3K or MAPK, may strongly activate NF-κB (p100/p52) expression [52]. In this perspective, PIM2 triggered significant p65 NF-κB translocation from the cytosol to the nucleus (Figure 6) in a Notch-PI3K-MAPK dependent manner. In order to further validate the role of Notch and NF-κB, bioinformatics analysis of mouse COX-2 and MMP-9 promoters [53] was carried out and analysis predicted the binding site for NF-κB between −507 to −519 bp and for RBP-Jk between −717 to −731 bp relative to the +1 transcription start site for COX-2 promoter. In this context, Chromatin immuno precipitation (ChIP) assays were carried out and the data clearly suggested that PIM2 treatment increased binding of NF-κB and RBP-Jk to COX-2 and MMP-9 promoter (Figure S15 and data not shown). These results further support the earlier observation that both RBP-Jk/CSL and NF-κB exerted positive regulatory roles during PIM2 induced COX-2 and MMP-9 expression (Figure 3G and Figure 6E–F). Our data from experiments on PIM2 induced nuclear translocation of NF-κB from cytosol in the presence of Notch or PI3K or MAPK activation inhibitors clearly suggest the possibility of frequent cross-talk between the Notch1 and PI3K-MAPK signaling pathways. Taken together, as depicted schematically in Figure 8, current investigation implicate PI3K and Notch1 signaling as obligatory early proximal signaling events during PIM2 induced COX-2 and MMP-9 expression in macrophages.

## Materials and Methods

### Mice and Cell Culture

C57BL/6 and C3H/HeJ mice were maintained at Central Animal Facility, Indian Institute of Science, Bangalore, India. In order to obtain peritoneal macrophages, peritoneal cavity of mice were injected with thioglycollate and on fourth day of injection, peritoneal macrophages were obtained by flushing peritoneal cavity with 10 ml ice-cold PBS and then cultured in DMEM (Sigma-Aldrich, USA) supplemented with L-glutamine and 10% Fetal Bovine Serum (FBS) (Sigma-Aldrich, USA). The experiments with macrophages derived from mice were carried out after the approval from the Indian Institute of Science Institutional Animal Ethics Committee in accordance with Government of India Guidelines as well as from Institutional Biosafety Committee of Indian Institute of Science, Bangalore, India. RAW 264.7 mouse macrophage cell line (Obtained from National Centre for Cell Sciences, Pune, India) was cultivated in DMEM supplemented with L-glutamine and 10% FBS.

### Preparation of PIM2 fraction

Phosphatidyl-*myo*-inositol mannosides-2 (PIM2) have been purified from *M. bovis* BCG 1173P2 (the Pasteur strain) envelope, on QMA-Spherosil M (BioSepra SA, Villeneuve-la-Garenne, France) column, as previously described [24].

### Chemicals and reagents

General laboratory reagents were purchased from Sigma-Aldrich, USA or Merck, Germany. Anti-Ser473 AKT, anti-AKT, anti-Thr70 4EBP1, anti-4EBP1, anti-Thr180/Tyr182 p38 MAPK, anti-p38 MAPK, anti-Thr202/Tyr204 pERK1/2, anti-ERK1/2, anti-Ser338 Raf, anti-Raf, anti-Ser536 NF-κB, anti-NF-κB, anti-Ser32 IκBα and anti-Cleaved Notch1 antibodies were purchased from Cell Signaling Technology, USA. Anti-Notch1 and anti-RBP-Jk antibodies were purchased from Santa Cruz Biotechnology, USA. Anti-COX-2, anti-PCNA and HRP conjugated anti- mouse IgG antibodies were obtained from Calbiochem, USA. Anti-β-actin and anti-MMP-9 antibodies were obtained from Sigma-Aldrich, USA. Anti-CD44 and anti-CD16/32 (Fc blocker) antibodies were purchased from eBioscience, USA. Cy2 or Cy5 or HRP conjugated anti-rat and anti-rabbit IgG antibodies were purchased from Jackson Immunoresearch, USA. Purified PGE2 was purchased from Sigma-Aldrich, USA.

### Treatment with pharmacological inhibitors

The pharmacological inhibitors were obtained from Calbiochem, USA and were reconstituted in sterile DMSO and used at following concentrations. LY294002 (50 µM), AKT Inhibitor II (AKT I-II, 10 µM), γ-secretase inhibitor I (GSI-I) (10 µM), SB203580 (20 µM), U0126 (10 µM), SP600125 (50 µM), Manumycin (20 µM), NS-398 (10 µM). DMSO at 0.1% concentration was used as the vehicle control. In all experiments with inhibitors, a tested concentration was used after careful titration experiments assessing the viability of the macrophages. In experiments with inhibitors, the macrophages were treated with a given inhibitor for 60 minutes before treatment with PIM2. We had tested different concentrations of various inhibitors used in the current study on their efficacy in terms of inhibition of a given kinase molecule. For example, U0126, a known inhibitor for MEK1/2 (which activates ERK1/2) significantly blocked *M. bovis* BCG triggered ERK1/2 phosphorylation (Figure S16A). Similarly, SB203580 abrogated PIM2 triggered IκBα phosphorylation (Figure S16B).

### Stable transfection of Notch Intracellular Domain (NICD)

RAW 264.7 stable cell line expressing NICD was generated by transfection with 2 µg of plasmid pCMV-NICD using Lipofectamine™ 2000 (Invitrogen, USA). After 48 h, cells were cultured for 10–12 days in DMEM complete medium supplemented with 400 µg /ml G418 (Calbiochem, USA) for selective growth of cells transfected with plasmid. To use as mock control RAW 264.7 cells stably transfected with plasmid pCMV were also generated. Stable expression of NICD in transfected cells was confirmed by analyzing protein level of NICD by western blotting and Hes 1 mRNA expression, a transcriptional target for Notch1 by quantitative real time PCR.

### siRNA transfections

RAW 264.7 cells were transfected with 100 nM siRNA using Lipofectamine™ 2000 (Invitrogen, USA) according to manufacturer's instructions. Transfection efficiency has been more than 50% through all the experiments as determined by counting the number of siGLO Lamin A/C positive cells in a microscopic field using fluorescent microscope. 96 h post transfection, the cells were treated with 4 µg/ml PIM2 for 12 h. Later total cell lysates were prepared and analyzed for indicated proteins by western blotting. siRNAs specific to Notch1, CSL/RBP-Jk, AKT, control siRNA and siGLO Lamin A/C siRNA were obtained from Dharmacon, USA as siGENOME™ SMARTpool reagent, which contains a pool of four different double-stranded RNA oligonucleotides (siRNA). Further, in all experiments involving siRNA, the specificity of each siRNA was assessed showing that an irrelevant gene like Actin was not suppressed as well as the relevant one being suppressed.

### Transient transfections and luciferase assays

RAW 264.7 cells were transfected with promoter constructs of MMP-9 (MMP-9 Luc) and Hes1 (Hes1 Luc) using Polyethylenimine (PEI, Sigma-Aldrich, USA). After 36 hrs of transfection, cells were treated with PIM2 for 12 h. Cells were harvested and lysed in reporter lysis buffer (Promega, USA) and luciferase activity was assayed using Luciferase assay reagent (Promega, USA). The results were normalized for transfection efficiencies by assay of β-galactosidase activity.

### Quantitative real Time PCR

Τotal RNA was isolated from cells using TRIzol (Sigma-Aldrich, USA) following to manufacturer's protocol and then treated with RNase free DNase (Promega, USA) to remove any potential DNA contamination. For RT-PCR, 2 µg of total RNA was converted into cDNA using First strand cDNA synthesis kit (Fermentas, USA). Quantitative real time PCR was performed with SYBR Green PCR mix from Finnzymes, Finland. All reactions were repeated at least three times independently to ensure the reproducibility of the results. Amplification of housekeeping gene GAPDH was used as internal control. The forward and reverse primer pairs used were as follows: GAPDH forward 5′-gagccaaacgggtcatcatct-3′, GAPDH reverse 5′-gaggggccatccacagtctt-3′, COX-2 forward 5′- gtatcagaaccgcattgcctc-3′, COX-2 reverse 5′- cggcttccagtattgaggagaacagat-3′, MMP-9 forward 5′-gagctgtgcgtcttccccttc-3′, MMP-9 reverse 5′-ggaatgatctaagcccagtgc-3′ Notch1 forward 5′- agaatggcatggtgcccag-3′, reverse 5′- tggtggagaggctgctgtgtag- 3′, Notch2 forward 5′- gatggaggtgactgttccctca -3′, reverse 5′- cgtcttgctattcctctggcac- 3′, Notch3 forward 5′- gatttcccatacccacttcgg-3′, reverse 5′- tgtgtaatgcaaaaccctcagg- 3′, Notch4 forward 5′- gttgaagaattgatcgcagcc -3′, reverse 5′- aggaaaagcggcgtctgtt- 3′, Jagged1 forward 5′- agaagtcagagttcagaggcgtcc -3′, reverse 5′- agtagaaggctgtcaccaagcaac- 3′, Jagged 2 forward 5′- tgctgtggaggtggctatgtct-3′, reverse 5′- tgtttccaccttgacctcggt- 3′, Dll 1 forward 5′- ggacctcagtgagaggcatatgg -3′, reverse 5′- ggcaattggctaggttgttcatg- 3′, Dll 3 forward 5′- agttgcacttctcctaccgcg -3′, reverse 5′- acggcattcatcaggctcttc- 3′, Dll4 forward 5′- gtgaactgcacatcagcgattg -3′, reverse 5′- gttgcagacgaagttgtttggg- 3′, Lunatic Fringe forward 5′- gacgtgtacatcggcaagcc -3′, reverse 5′- agccaatggtgcagtcatcg- 3′, Manic Fringe forward 5′- ggctacatcatcgagtgcaagc -3′, reverse 5′- aggagacaatggagggagcg- 3′, Radical Fringe forward 5′- ctgtgttgctgctactgccg -3′, reverse 5′- ctcgtgagatccaggtacgc- 3′, Kurzbanian forward 5′- ggccagcctatctgtggaaac -3′, reverse 5′- gtacagcagggtccttgactcg- 3′, Presinilin forward 5′- gacagtggttctgggaacgatg -3′, reverse 5′- cctccgggtcttcactcgttag- 3′, Hes 1 forward 5′- gagaggctgccaaggtttttg -3′, reverse 5′- cactggaaggtgacactgcg - 3′. All the primers were purchased from Sigma Genosys, India.

### Preparation of cell lysate and western blot analysis

Cells were washed with ice cold PBS and scrapped off the culture dish and collected by centrifugation. Cell pellets were lysed in RIPA buffer constituting 50 mM Tris-HCl, pH 7.4, 1% NP-40, 0.25% Sodium deoxycholate, 150 mM NaCl, 1 mM EDTA, 1 mM PMSF, 1 µg/ml of each aprotinin, leupeptin, pepstatin, 1 mM Na_3_VO_4_, 1 mM NaF and incubated on ice for 30 min. Whole cell lysate was collected by centrifuging lysed cells at 13000 rpm , 10 min at 4°C. Protein concentration in each cell lysate was determined by Bradford's method of protein detection. Equal protein amount from each cell lysate was subjected to SDS-PAGE and transferred onto polyvinylidene difluoride membranes (Millipore, USA) by the semidry Western blotting (Bio-Rad, USA) method. Nonspecific binding was blocked with 5% nonfat dry milk powder in TBST (20 mM Tris-HCl (pH 7.4), 137 mM NaCl, and 0.1% Tween 20) for 60 min. The blots were incubated overnight at 4°C with primary antibodies diluted in TBST with 5% BSA. After washing with TBST, blots were incubated with anti-rabbit or anti-mouse IgG secondary Ab conjugated to HRP (Jackson Immunoresearch, USA) diluted in TBST with 5% BSA for 2 h. After further washing in TBST, the immunoblots were developed with the ECL system (Perkin Elmer, USA) following the manufacturer's instructions.

### Nuclear and cytosolic fractionation of peritoneal macrophages

Peritoneal macrophages were grown in 60-mm dishes and treated as indicated. After treatment, cells were harvested by centrifugation at 13000 rpm for 5 min and cell pellets were washed with phosphate buffered saline (PBS) and gently resuspended in Buffer A (10 mM HEPES pH 7.9, 10 mM KCl, 0.1 mM EDTA, 0.1 mM EGTA, 1 mM DTT and 0.5 mM PMSF). After incubation on ice for 15 min, cell membrane was disrupted with 10% NP-40 and the nuclear pellets were recovered by centrifugation 13000 rpm for 15 min at 4°C and supernatant was used as cytosolic extract. Nuclear pellets were lysed with Buffer C (20 mM HEPES pH7.9, 0.4 M NaCl, 1 mM EDTA, 1 mM EGTA, 1 mM DTT and 1 mM PMSF) and nuclear extracts were collected after centrifugation at 13000 rpm for 20 min at 4°C.

### Immunofluorescence

Resident mouse peritoneal macrophages were seeded on to coverslips and treated as indicated. The cells were fixed with 3% formaldehyde for 10 min at room temperature. Primary antibodies were incubated for 1 h at room temperature following three washes with PBS. Secondary antibodies (Cy2 conjugated anti-rabbit IgG or Cy5 conjugated anti-rat IgG) were incubated in the dark for 1 h at room temperature and then washed three times with PBS. Coverslips with cells were mounted on a slide with fluoromount G. Confocal images were taken on Zeiss LSM 510 Meta confocal laser scanning microscope using plan –Apochromat 63X /1.4 oil DIC objective and Argon/2 458, 477, 488, 514 nm and HeNe 543 lasers. During colocalization studies, a series of images at an interval of 0.37-mm focal planes were collected into a z-stack to ascertain the actual colocalization. Every single layer of z-stack was subjected to image analysis by LSM 5 image examiner software to visualize and locate protein expression.

### Flow cytometry analysis

The cells were washed with PBS and were fixed in 0.1% formaldehyde. In order to avoid nonspecific binding, cells were incubated with 0.5 µg Fc blocker (per 10^6^ cells) for 30 minutes on ice followed by staining with rabbit anti-mouse MMP-9. This was followed by staining with flouro isothiocyanate (FITC) or Cy2 conjugated anti-rabbit IgG. Similarly, cells were labeled with rat anti-mouse CD44 antibody followed by Cy5 conjugated anti-rat IgG and were analyzed using FACScan (BD Biosciences, USA). Dead cells were excluded from the analysis by their forward and side-way light-scattering properties.

### Inhibition of PIM2 synthesis in *M. bovis* BCG

*M. bovis* BCG Pasteur 1173P2 was grown to mid log phase in Souton's medium and treated with D- Mannosamine (ManN) (Sigma Aldrich, USA) at a concentration of 5 mg/ml. Cultures were centrifuged after 24 h and aliquots were frozen at −70°C. Representative vials were thawed and enumerated for viable colony forming unit on Middlebrook 7H10 agar (Difco, USA) supplemented with OADC (oleic acid, albumin, dextrose, catalase). For macrophage infections, single cell suspension of bacteria were obtained after short pulses of sonication and macrophages were infected at MOI of 1∶1. Further, in experiments with D-mannosamine, a tested concentration was chosen after careful titration experiments assessing the viability (CFU) of *M. bovis* BCG. The inhibition of PIM2 synthesis was confirmed by the Thin-Layer chromatography of cell wall lipids as described [21]. Briefly, Bacilli were washed extensively in 0.05% Tween 80/PBS followed by a detergent-free wash before extraction in chloroform and methanol. The bacterial pellet was extracted twice in chloroform/methanol (2∶1 v/v) with sonication. Bacterial debris was removed by centrifugation, and hydrophilic contaminants were removed using a Folch wash. TLC analysis of lipids was performed on aluminum backed plates of silica gel 60 F254 (Merck, Germany). The solvent used was CHCl_3_/CH_3_OH/NH_4_OH/H_2_O (65∶24∶0.4∶3.6 v/v). Visualization of resolved lipids was achieved with a charring spray (10% CuSO4 in 8% phosphoric acid) and heating at 110°C.

### Chromatin Immunoprecipitation Assay

Chromatin immunoprecipitation (ChIP) assays were carried out following a protocol provided by Upstate Biotechnology, USA with modifications. Peritoneal macrophages were left untreated or treated with PIM2 for 6 h as in the case of NF-κB or 12 h for CSL/RBP-Jk. The cells were fixed with 1.42% formaldehyde for 15 min at room temperature followed by inactivation of formaldehyde with addition of 125 mM glycine. Chromatin extracts containing DNA fragments with an average size of 500 bp were immunoprecipitated using anti NF-κB for NF-κB ChIP or anti-Notch1 antibody for RBP-Jk ChIP. Purified DNA was analyzed by quantitative PCR by using the SYBR Green method (Finnzymes). Regions with the NF-κB and RBP-Jk binding site in the mouse COX-2 promoter were amplified using primer pairs, NF-κB forward, 5′-ttaaccggtagctgtgtgcgt- 3′, reverse, 5′-tctccggtttcctcccagt-3′; and RBP-Jk forward, 5′-tcccgtgaaaagagttgctga-3′, reverse, 5′-ttcatggaactaccctccgg- 3′. The binding sites in MMP-9 promoter were amplified with primers NF-κB forward, 5′- gcgacaaagggtctgtttttg- 3′, reverse, 5′- tgactcagcttcctctcctgg-3′; and RBP-Jk forward, 5′-atcagtcagggccgtcagac-3′, reverse, 5′- gacccacaggaaaccacagaac- 3′. 28S rRNA was used as control in the PCR and the primers were forward, 5′-ctgggtataggggcgaaagac-3′ and reverse, 5′-ggccccaagacctctaatcat-3′. All results were normalized either by respective input values or by amplification of 28 S rRNA. All ChIP experiments were repeated at least three times.

### Statistical analysis

Levels of significance for comparison between samples were determined by the Student *t* test distribution. The data in the graphs is expressed as the mean±SEM. Graphpad Prism 3.0 software (Graphpad software, USA) was used for all the statistical analysis.

## Supporting Information

Figure S1 (0.08 MB DOC)

Figure S2 (0.05 MB DOC)

Figure S3 (0.05 MB DOC)

Figure S4 (0.03 MB DOC)

Figure S5 (0.05 MB DOC)

Figure S6 (0.05 MB DOC)

Figure S7 (0.03 MB DOC)

Figure S8 (0.06 MB DOC)

Figure S9 (0.03 MB DOC)

Figure S10 (0.06 MB DOC)

Figure S11 (0.22 MB DOC)

Figure S12 (0.04 MB DOC)

Figure S13 (0.15 MB DOC)

Figure S14 (0.09 MB DOC)

Figure S15 (0.03 MB DOC)

Figure S16 (0.09 MB DOC)
