# Supplementary material for: PIM2 Induced COX-2 and MMP-9 Expression in Macrophages Requires PI3K and Notch1 Signaling
Source: PLoS One. 2009 Mar 17;4(3):e4911. doi: 10.1371/journal.pone.0004911 (PMC2654112; doi:10.1371/journal.pone.0004911)
Supplement: Figure S1 — (0.08 MB DOC) [file pone.0004911.s001.doc]

**Figure S1**

**
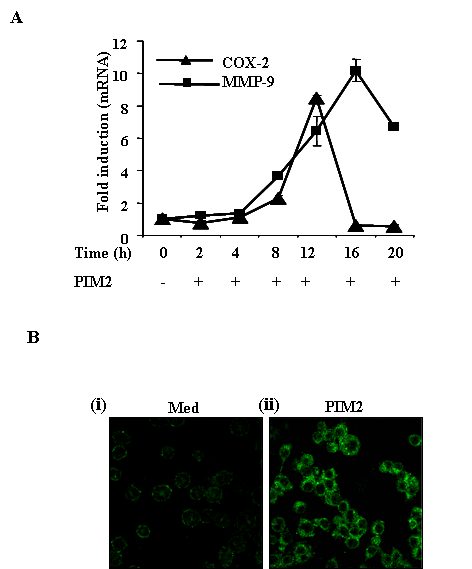
**

**Figure S1. Time kinetics of PIM2 triggered expression of COX-2 and MMP-9.** (A) Mouse Macrophages were treated with PIM2 for the time points as indicated and transcript levels of COX-2 and MMP-9 were analyzed by real-time PCR. (B). Macrophages were treated with 4.0μg/ml of PIM2 for 12h and surface MMP-9 expression was analyzed by confocal microscopy. MMP-9 expression was detected by binding of specific or isotype matched antibodies followed by probing with Cy-2 (green) labeled anti-rabbit secondary antibody. The data is representative of three independent experiments. *Med,* Medium*.*
