# Supplementary material for: PIM2 Induced COX-2 and MMP-9 Expression in Macrophages Requires PI3K and Notch1 Signaling
Source: PLoS One. 2009 Mar 17;4(3):e4911. doi: 10.1371/journal.pone.0004911 (PMC2654112; doi:10.1371/journal.pone.0004911)
Supplement: Figure S2 — (0.05 MB DOC) [file pone.0004911.s002.doc]

**Figure S2**


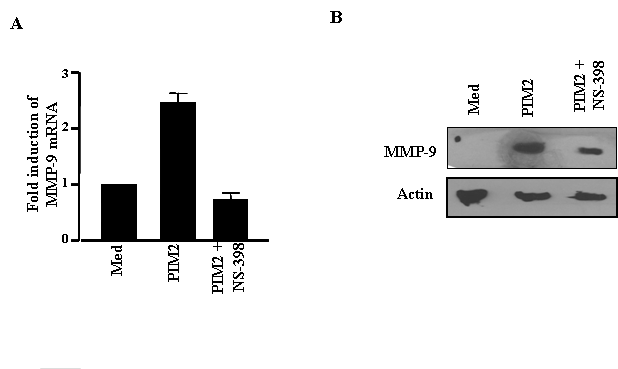


**Figure S2.** **COX-2 regulates PIM2 induced MMP-9 expression on macrophages** (A). Mouse macrophages were cultured with or with out NS-398 (10μM) and treated with 4.0μg/ml of PIM2 for 12 h. The mRNA levels were analyzed by real time PCR as well as (B). protein levels of MMP-9 were analyzed by immunoblotting. The data presented in the figure represents three independent experiments. *Med*, Medium.
