# Supplementary material for: PIM2 Induced COX-2 and MMP-9 Expression in Macrophages Requires PI3K and Notch1 Signaling
Source: PLoS One. 2009 Mar 17;4(3):e4911. doi: 10.1371/journal.pone.0004911 (PMC2654112; doi:10.1371/journal.pone.0004911)
Supplement: Figure S3 — (0.05 MB DOC) [file pone.0004911.s003.doc]

**Figure S3**


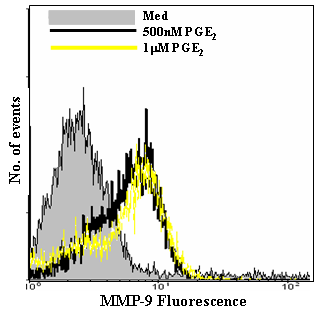


**Figure S3.** **PGE2 triggers surface expression of MMP-9.** Macrophages were treated with different concentrations of PGE2 and cell surface expression of MMP-9 was analyzed by flow cytometry using rabbit anti-MMP-9 antibody followed by probing with Cy-2 labeled anti-rabbit secondary antibody. The data presented in the figure is representative of two independent experiments. *Med*, Medium.
