# Supplementary material for: PIM2 Induced COX-2 and MMP-9 Expression in Macrophages Requires PI3K and Notch1 Signaling
Source: PLoS One. 2009 Mar 17;4(3):e4911. doi: 10.1371/journal.pone.0004911 (PMC2654112; doi:10.1371/journal.pone.0004911)
Supplement: Figure S4 — (0.03 MB DOC) [file pone.0004911.s004.doc]

**Figure S4**

**Figure S4.** **Direct activation of COX-2 and MMP-9 expression by PIM2.** Macrophages were treated with PIM2 with or without prior treatment with cycloheximide for 12hr and (A) COX-2 or (B) MMP-9 mRNA levels were analyzed by real-time PCR. *Med*, Medium.
