# Supplementary material for: PIM2 Induced COX-2 and MMP-9 Expression in Macrophages Requires PI3K and Notch1 Signaling
Source: PLoS One. 2009 Mar 17;4(3):e4911. doi: 10.1371/journal.pone.0004911 (PMC2654112; doi:10.1371/journal.pone.0004911)
Supplement: Figure S5 — (0.05 MB DOC) [file pone.0004911.s005.doc]

**Figure S5**


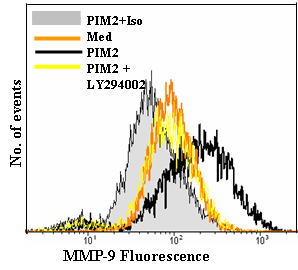


**Figure S5.** **PIM2 triggered cell-surface expression of MMP-9 requires the participation of PI3 Kinase pathway.** Cell-surface MMP-9 expression was analyzed by flow cytometry on PIM2 treated macrophages that were cultured in the presence or absence of LY294002 (50 μM) or 0.1% DMSO as vehicle control. Data in the figure is representative of two independent experiments. *Med*, Medium.
