# Supplementary material for: PIM2 Induced COX-2 and MMP-9 Expression in Macrophages Requires PI3K and Notch1 Signaling
Source: PLoS One. 2009 Mar 17;4(3):e4911. doi: 10.1371/journal.pone.0004911 (PMC2654112; doi:10.1371/journal.pone.0004911)
Supplement: Figure S6 — (0.05 MB DOC) [file pone.0004911.s006.doc]

**Figure S6**


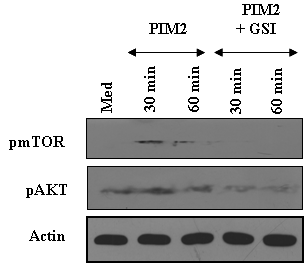


**Figure S6. PIM2 induced activation of PI3 kinase pathway is dependent on Notch signaling.** Mouse macrophages were pretreated with GSI-I and activation of mTOR and AKT at 30 and 60 min post treatment of PIM2 was analyzed by immunoblotting. The blots are representative of two independent experiments. *Med*, Medium.
