# Supplementary material for: PIM2 Induced COX-2 and MMP-9 Expression in Macrophages Requires PI3K and Notch1 Signaling
Source: PLoS One. 2009 Mar 17;4(3):e4911. doi: 10.1371/journal.pone.0004911 (PMC2654112; doi:10.1371/journal.pone.0004911)
Supplement: Figure S7 — (0.03 MB DOC) [file pone.0004911.s007.doc]

**Figure S7**


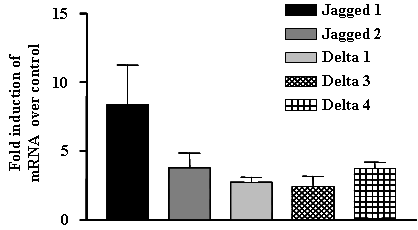


**Figure S7.** **PIM2 triggers Notch1 activation as well as expression of Notch ligands.** Real time PCR analysis of mRNA levels of Jagged1, Jagged2, Dll 1, Dll3 and Dll 4 in macrophages treated with PIM2.
