# Supplementary material for: PIM2 Induced COX-2 and MMP-9 Expression in Macrophages Requires PI3K and Notch1 Signaling
Source: PLoS One. 2009 Mar 17;4(3):e4911. doi: 10.1371/journal.pone.0004911 (PMC2654112; doi:10.1371/journal.pone.0004911)
Supplement: Figure S8 — (0.06 MB DOC) [file pone.0004911.s008.doc]

**Figure S8**


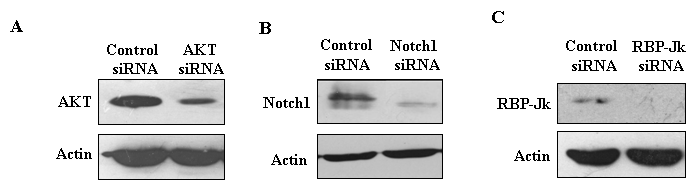


**Figure S8.** RAW 264.7 cells were transfected with either control siRNA or siRNA directed to AKT (A) or Notch1 (B) or RBP-Jk (C). Three days post transfection, protein levels of AKT, or Notch1 or RBP-Jk were analyzed by immunoblotting.
