# Supplementary material for: PIM2 Induced COX-2 and MMP-9 Expression in Macrophages Requires PI3K and Notch1 Signaling
Source: PLoS One. 2009 Mar 17;4(3):e4911. doi: 10.1371/journal.pone.0004911 (PMC2654112; doi:10.1371/journal.pone.0004911)
Supplement: Figure S9 — (0.03 MB DOC) [file pone.0004911.s009.doc]

**Figure S9**


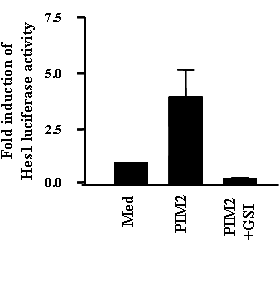


**Figure S9. Inhibition of Notch activation abrogates RBP-Jk dependent Hes1 transcription.** RAW 264.7 macrophages were transfected with Hes1-Luc, treated with GSI-I (10 μM) or 0.1% DMSO followed by treatment with PIM2. The Hes1 promoter activity was evaluated by Luciferase assay. The data presented in the figure is representative of three independent experiments. *Med*, Medium.
