# Supplementary material for: PIM2 Induced COX-2 and MMP-9 Expression in Macrophages Requires PI3K and Notch1 Signaling
Source: PLoS One. 2009 Mar 17;4(3):e4911. doi: 10.1371/journal.pone.0004911 (PMC2654112; doi:10.1371/journal.pone.0004911)
Supplement: Figure S10 — (0.06 MB DOC) [file pone.0004911.s010.doc]

**Figure S10**


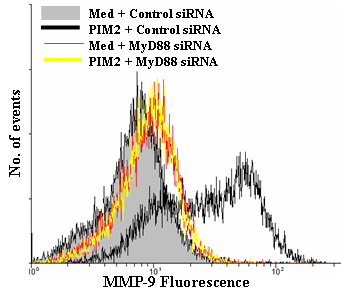


**Figure S10.** **MyD88 is involved in PIM2 induced MMP-9 expression.** RAW264.7 macrophages were transfected with siRNA targeted to MyD88 or with control siRNA. Three days post transfection, cells were treated with PIM2 and cell surface MMP-9 expression was analyzed by flow cytometry. Data in the figure is representative of three independent experiments. *Med*, Medium.
