# Supplementary material for: PIM2 Induced COX-2 and MMP-9 Expression in Macrophages Requires PI3K and Notch1 Signaling
Source: PLoS One. 2009 Mar 17;4(3):e4911. doi: 10.1371/journal.pone.0004911 (PMC2654112; doi:10.1371/journal.pone.0004911)
Supplement: Figure S11 — (0.22 MB DOC) [file pone.0004911.s011.doc]

**Figure S11**

**
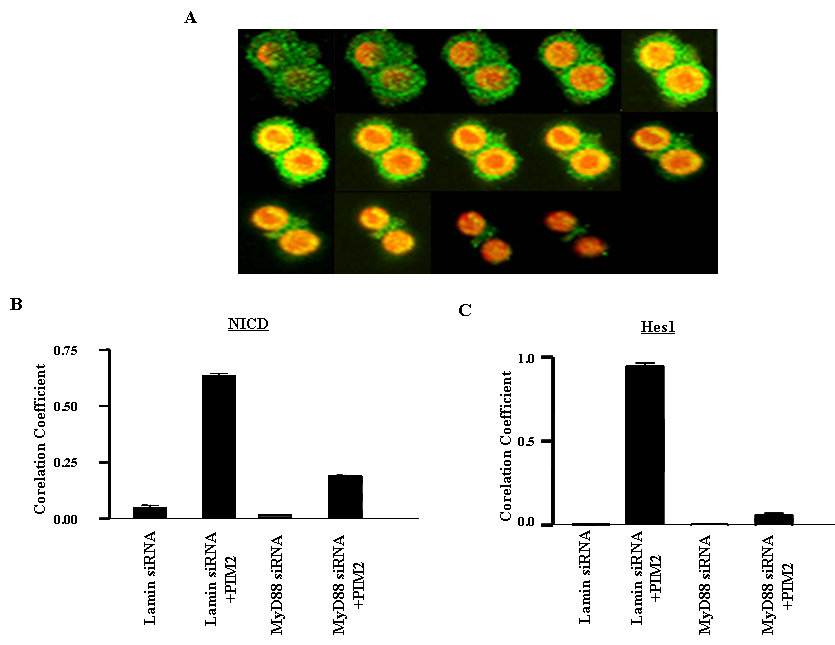
**

**Figure S11. Requirement of MyD88 in PIM2 triggered nuclear translocation of Hes1 and activated Notch1, NICD.** PIM2 triggered nuclear translocation of Hes1 (A & C) and NICD (B) which could be blocked by siRNA to MyD88. A series of images at an interval of 0.37-mm interval focal planes were collected into a z-stack and every single layer of z-stack was subjected to image analysis by LSM 5 image examiner software to visualize and locate Hes1 (A) or NICD protein expression in the nucleus. Colocalization correlation coefficient for (B) NICD or (C) Hes1 was generated from scatter plots derived from numerical analysis on 15 cells in each group and the results are representative of three independent experiments.
