# Supplementary material for: PIM2 Induced COX-2 and MMP-9 Expression in Macrophages Requires PI3K and Notch1 Signaling
Source: PLoS One. 2009 Mar 17;4(3):e4911. doi: 10.1371/journal.pone.0004911 (PMC2654112; doi:10.1371/journal.pone.0004911)
Supplement: Figure S12 — (0.04 MB DOC) [file pone.0004911.s012.doc]

**Figure S12**

**
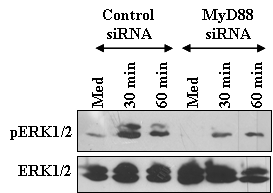
**

**Figure S12.** **MyD88 is involved in PIM2 mediated activation of ERK1/2 MAP kinase.** RAW264.7 macrophages were transfected with siRNA targeted to MyD88 or with control siRNA. Three days post transfection, cells were treated with PIM2 for the indicated time points and activation of ERK1/2 was assessed by immunoblotting. The data presented in the figure is representative of two independent experiments. *Med*, Medium.
