# Supplementary material for: PIM2 Induced COX-2 and MMP-9 Expression in Macrophages Requires PI3K and Notch1 Signaling
Source: PLoS One. 2009 Mar 17;4(3):e4911. doi: 10.1371/journal.pone.0004911 (PMC2654112; doi:10.1371/journal.pone.0004911)
Supplement: Figure S13 — (0.15 MB DOC) [file pone.0004911.s013.doc]

**Figure S13.**


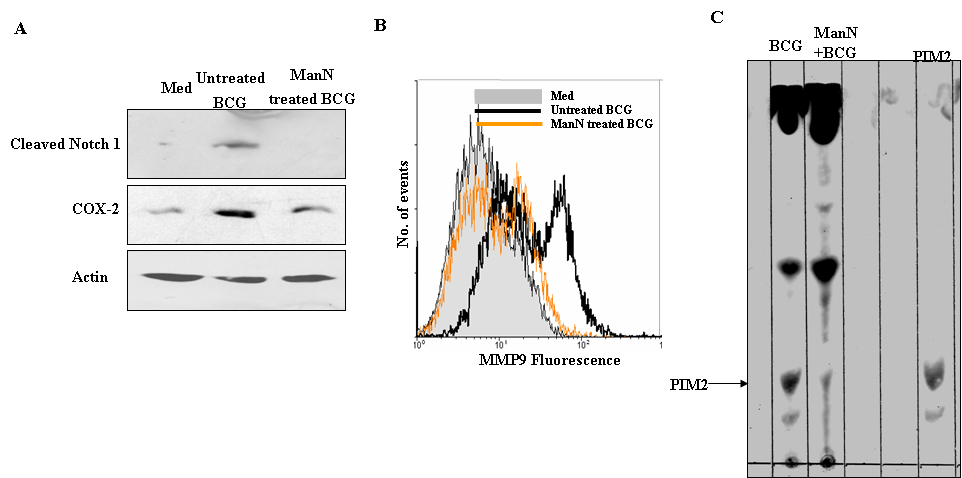


**Figure S13**. **Inhibition of pimB, a key enzyme involved in synthesis of PIM2 in *M. bovis* BCG by D-Mannosiamine (ManN) markedly reduces the expression of activated Notch1 (NICD), COX-2 or MMP-9.** (A)*.* Mouse macrophages were infected either with untreated or ManN treated *M. bovis* BCG and generation of NICD as well as COX-2 levels were evaluated by immunoblotting and (B). surface expression of MMP-9 was evaluated by flow cytometry. (C). Cell wall associated lipids were extracted from untreated or ManN treated *M. bovis* BCG and analyzed by thin-layer chromatography. PIM2 was loaded as standard. The data presented in the figure is representative of two independent experiments. *Med*, Medium.
