# Supplementary material for: PIM2 Induced COX-2 and MMP-9 Expression in Macrophages Requires PI3K and Notch1 Signaling
Source: PLoS One. 2009 Mar 17;4(3):e4911. doi: 10.1371/journal.pone.0004911 (PMC2654112; doi:10.1371/journal.pone.0004911)
Supplement: Figure S14 — (0.09 MB DOC) [file pone.0004911.s014.doc]

**Figure S14**

**
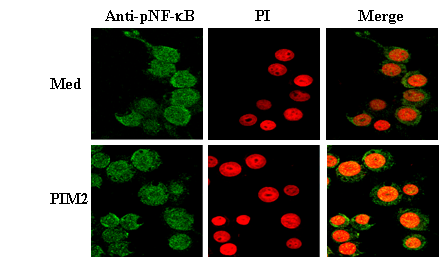
**

**Figure S14. PIM2 triggered nuclear translocation of activated form of NF-κB.** Mouse peritoneal macrophages were treated with 4.0μg/ml of PIM2 for 60 min and nuclear translocation of phosphorylated form of p65 NF-κB was analyzed by confocal microscopy. The image presented in the figure is representative of two independent experiments. *Med*, Medium; *PI,* Propidium Iodide.
