# Supplementary material for: PIM2 Induced COX-2 and MMP-9 Expression in Macrophages Requires PI3K and Notch1 Signaling
Source: PLoS One. 2009 Mar 17;4(3):e4911. doi: 10.1371/journal.pone.0004911 (PMC2654112; doi:10.1371/journal.pone.0004911)
Supplement: Figure S15 — (0.03 MB DOC) [file pone.0004911.s015.doc]

**Figure S15**

**
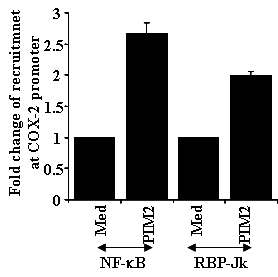
**

**Figure S15. NF-κB and RBP-Jk/CSL are recruited to the COX-2 promoter *in vivo.*** Recruitment of NF-κB and RBP-Jk/CSL at the COX-2 promoter was analyzed by the chromatin immunoprecipitation assay with antibodies to NF-κB or Notch1 respectively in PIM2 treated cell lysates. The recruitment of NF-κB or Notch1 complexed with CSL/RBP-Jk was assessed by real time PCR. The data presented in the figure is representative of three independent experiments. *Med*, Medium.
