# Supplementary material for: PIM2 Induced COX-2 and MMP-9 Expression in Macrophages Requires PI3K and Notch1 Signaling
Source: PLoS One. 2009 Mar 17;4(3):e4911. doi: 10.1371/journal.pone.0004911 (PMC2654112; doi:10.1371/journal.pone.0004911)
Supplement: Figure S16 — (0.09 MB DOC) [file pone.0004911.s016.doc]

**Figure S16**

**
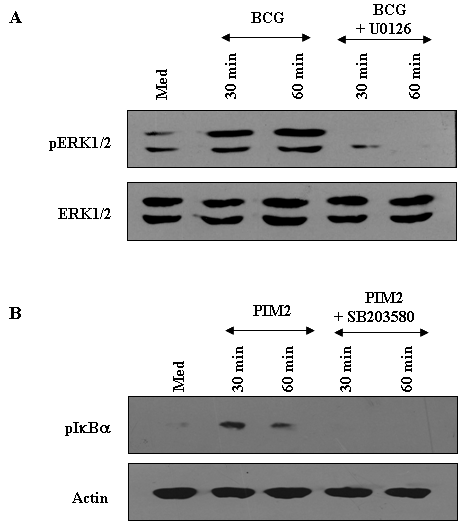
**

**Figure S16. Inhibition of an intended target signaling molecule by specific pharmacological inhibitor**. (A). Decrease in *M. bovis* BCG induced activation of ERK1/2 by U0126 (10μM) (B). Suppression of PIM2 induced phosphorylation of IκBα by SB203580 (20μM). The data presented in the figure is representative of two independent experiments. *Med*, Medium.
